# Industrial production, application, microbial biosynthesis and degradation of furanic compound, hydroxymethylfurfural (HMF)

**DOI:** 10.3934/microbiol.2018.2.261

**Published:** 2018-03-21

**Authors:** Yu Wang, Caroline A. Brown, Rachel Chen

**Affiliations:** 1 Department of Chemistry and Biochemistry, University of North Georgia-Dahlonega, Dahlonega, GA, 30597, USA; 2 School of Chemical & Biomolecular Engineering, Georgia Institute of Technology, Atlanta, GA 30332, USA

**Keywords:** furan-containing compound, 4-HMF, 5-HMF, HMF metabolism, HMF biosynthesis, HMF degradation

## Abstract

Biorefinery is increasingly embraced as an environmentally friendly approach that has the potential to shift current petroleum-based chemical and material manufacture to renewable sources. Furanic compounds, particularly hydroxymethylfurfurals (HMFs) are platform chemicals, from which a variety of value-added chemicals can be derived. Their biomanufacture and biodegradation therefore will have a large impact. Here, we first review the potential industrial production of 4-HMF and 5-HMF, then we summarize the known microbial biosynthesis and biodegradation pathways of furanic compounds with emphasis on the enzymes in each pathway. We especially focus on the structure, function and catalytic mechanism of MfnB (4-(hydroxymethyl)-2-furancarboxyaldehyde-phosphate synthase) and hmfH (HMF oxidase), which catalyze the formation of phosphorylated 4-HMF and the oxidation of 5-HMF to furandicarboxylic acid (2,5-FDCA), respectively. Understanding the structure-function relationship of these enzymes will provide important insights in enzyme engineering, which eventually will find industry applications in mass-production of biobased polymers and other bulk chemicals in future.

## 1. Introduction of HMFs

With biorefinery becoming an industrial model, the search for platform chemicals to replace petroleum products with renewable raw materials has become important. The biorefinery model balances the needs of energy and economics resulting in the production of both low value biofuels and high value chemicals [1]. To help focus research efforts in the production of platform chemicals, the US Department of Energy released a Top “10 + 4” list of the most promising candidates for chemical production [2]. This list evaluated chemicals based on a set of criteria including properties like the compound receiving attention in literature, the compound having strong potential as a platform, and the product's ability to serve as a building block of biorefinery [1]. The list included chemicals such as glycerol, xylitol, aspartic acid, and furan-containing compounds. Heavy research efforts have been invested in refining the production process and using the furan-containing compounds [1].

**Figure 1.**
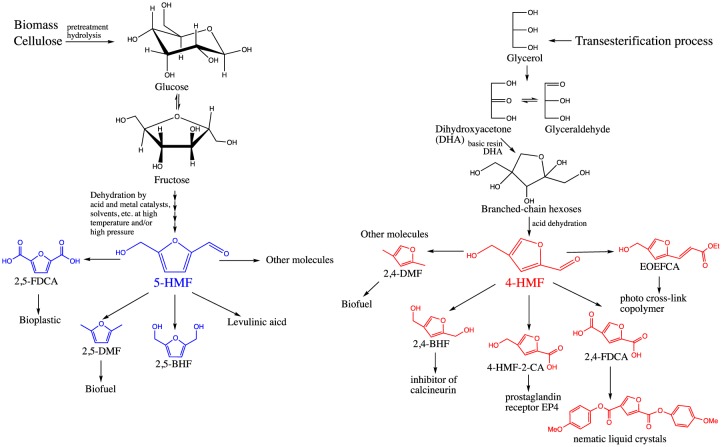
Synthesis and application of 5-HMF (left) [5],[7] and 4-HMF (right) [6].

Furfural and hydroxymethylfurfural (HMF) have the potential to be upgraded into numerous valuable chemicals [3]–[6]. 5-HMF, a rising star in recent years, has been identified as a versatile platform chemical for production of green polymers, pharmaceuticals, resins, solvents, fungicides and fuels [7],[8]. The chemical structure of 5-HMF is comprised of a furan ring, a hydroxyl group and a formyl group, which are the functional groups for oxidation-reduction, esterification or other reactions (Figure 1).

5-HMF as an intermediate molecule is important because it can be transformed through simple chemical reactions into a large number of useful compounds [5],[7],[9] (Figure 1, left). One of the most important applications of 5-HMF is to produce 2,5-dimethylfuran (2,5-DMF), the most valuable biofuel candidate, through catalytic hydrogenolysis of 5-HMF [10],[11]. 2,5-DMF stands out as an alternative biofuel due to its high octane number, high energy density, higher boiling point, lower water miscibility and lower volatility, which makes it a potential drop-in replacement to both gasoline and diesel [10],[11]. Studies have also shown that the combustion and emission properties of 2,5-DMF are comparable to that of commercial gasoline [12],[13]. In addition to DMF, hydroxymethylfuroic acid, alkomethylfurfurals, levulinic acid, furandicarboxylic acid (FDCA), bis(hydroxymethyl)furan (BHF) [3],[14] and the diether of 5-HMF, all have high potentials in fuel or polymer application [7]. Specifically, the physical properties of polyethylene 2,5-furandicarboxylic acid (PEF), a bio-based renewable plastic, are highly comparable to its analogue polyethylene terephthalate (PET) [15]–[20], a petroleum-derived plastic. In addition to PEF, however, there remains an increasing need for other bio-based diacids, which would find the potential in the development of other bio-based plastics.

4-HMF has the same functional groups and a similar structure as 5-HMF, but reports on 4-HMF production and application are very limited due to the difficulties to obtain in nature [4],[6]. 4-HMF can also be employed as a precursor molecule to produce 2,4-DMF or branched-chain alkanes as liquid transportation fuels [4]. Previous studies indicated that utilization of 4-HMF might find a broad application in the synthesis of 2,4-substituted furan compounds used in pharmaceutical and material industries [6] (Figure 1, right). 4-HMF could be upgraded into other valuable platform compounds such as 2,4-bis(hydroxymethyl)furan (2,4-BHF) (the precursor of calcineurin inhibitor [21]), 4-(hydroxymethyl)furan-2-carboxylic acid (4-HMF-2-CA) (which could convert to prostaglandin receptor EP4 [22]), furan-2,4-dicarboxylic acid (2,4-FDCA) (which could convert to liquid crystal materials) [23],[24], and (*E*)-5-(3-ethoxy-3-oxoprop-1-enyl)furan-3-carboxylic acid (EOEFCA) (the precursor of photo cross-linking of copolymer) [6].

## 2. Occurrence and industrial production of HMFs

HMF occurs naturally during the processing of many different foods including coffee, cereals, and vegetable products [25]. Furans are found at very low levels in groups of food that are rich in carbohydrates which contain hexoses that can be easily dehydrated to HMF [26], or amino acids which can react to form a Schiff base that further reacts to form HMF [25]. HMF occurs from the use of high temperatures during baking, roasting, or pasteurizing. The heat promotes the reaction of peptides and sugars into HMF. Coffee is one food product that has significant levels of HMF. The amount of HMF present in a coffee roast is directly correlated to the intensity of the heat used and a highly acidic environment during roasting supporting the idea the HMF is not naturally present in coffee but is instead a byproduct of the thermal processing [27].

HMF as a high-value product has drawn scientists' attention to developing an efficient process for the synthesis and bio-conversion of HMF to support future chemical and biofuel industries. Currently, most 5-HMFs are obtained from multiple step dehydration reactions of furanoses, which are derived from pretreatment and hydrolysis of cellulose and hemicellulose polymers [28]. The steps in the process of pretreating biomass substrates include using homogeneous mineral acid, Brønsted acidic ionic liquids (IL), Lewis acidic metal halides and recyclable heterogeneous catalysts in pure organic or aqueous solvents [29],[30] (Figure 1, left).

Studies on 4-HMF productions are very limited [4],[6]. Due to the complexities and difficulties of synthesis, 2,4-substituted furans were fairly expensive compounds [31]. The most recently published 4-HMF synthesis method starts with a base-catalyzed condensation of dihydroxyacetone (DHA), which is prepared from the oxidation of bio-mass derived glycerol through biocatalyzed or metal-catalyzed reactions [6]. The subsequent product ketohexoses are then converted to 4-HMF through an acid-catalyzed dehydration [6] (Figure 1, right). The steps in the process of synthesis require varieties of catalysts, controlling pH and air speed in reactors, and alternating temperatures (from 50 °C in oxidation step to ∼0 °C in condensation step and to 110 °C at dehydration step) [6].

## 3. Microbial biosynthesis of furanic compounds

**Figure 2.**
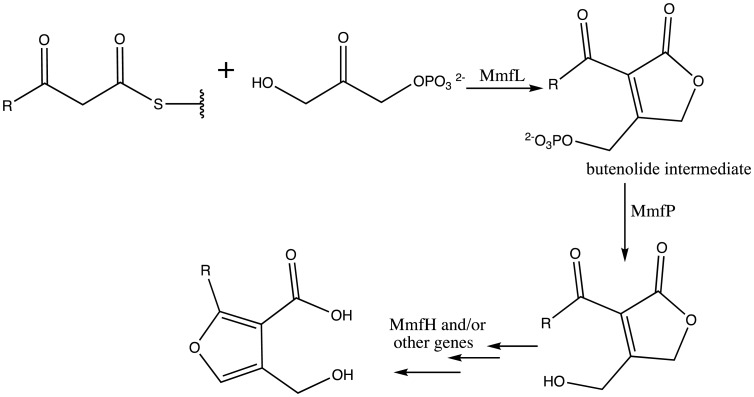
The hypothetical pathway for MMFs biosynthesis via butenolide intermediate [33].

The synthesis of furanic compounds occurs very rarely in biological systems. Few enzymes involved in the formation of furan-containing structures have been reported before. One example can be seen in the biosynthesis of the secondary metabolite, methylenomycin furans (MMFs) (contain a 4-hydroxymethylfuran-3-carboxylic acid core with various alkyl substituents at C2), which induce the production of antibiotics methylenomycin in *Streptomyces coelicolor* [32]. The gene products of *mmfL*, *mmfH*, *mmfP* are proposed to catalyze the formation of MMFs. In the proposed biosynthetic pathway, MmfL catalyzes the condensation of dihydroxyacetone phosphate and a β-ketoacyl-thioester intermediate in fatty acid biosynthesis likely through the butenolide intermediate, which then undergoes dephosphorylation, cyclization and dehydration reactions, likely catalyzed by MmfP and MmfH [32],[33] (Figure 2). The gene database annotates *mmfP* as a putative phosphatase. However, the precise functions of MmfP and MmfH have still to be established *in vitro* [33].

**Figure 3.**
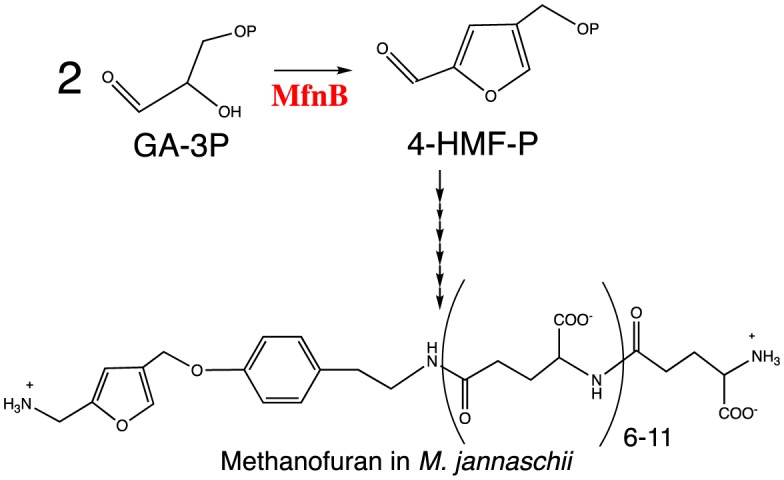
MfnB catalyzed reaction and the structure of methanofuran [34],[35].

**Figure 4.**
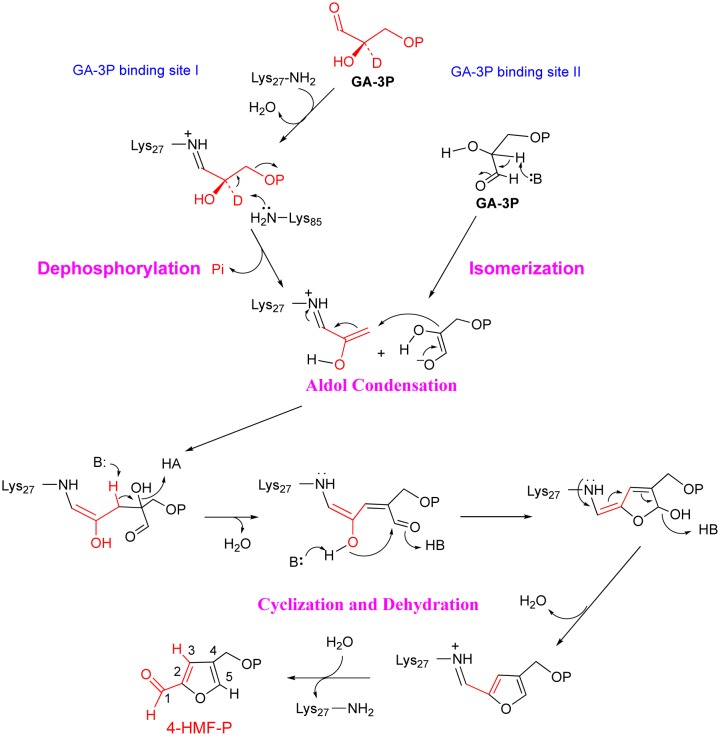
The proposed catalytic mechanism of MfnB [34].

Another example is a novel enzyme, 4-(hydroxymethyl)-2-furancarboxyaldehyde-phosphate synthase (MfnB) [34],[35]. MfnB catalyzes the formation of 4-hydroxymethylfurfural phosphate (4-HMF-P) from two molecules of D-glyceraldehyde-3phosphate (GA-3P) in the biosynthetic pathway to the furan moiety of the coenzyme methanofuran found in methanogens (Figure 3) [36]. The crystal structure of MfnB exhibits a typical α/β TIM barrel fold, which is commonly seen within Class I aldolases [37]–[44]. At the active site, two lysines and two aspartic acids are strictly conserved in both the MfnB and deoxyribose-5-phosphate aldolase (DERA, a class I aldolase) active site and are essential in the function of MfnB and DERA [45]. However, what is really amazing is the reactions catalyzed by MfnB are way beyond a simple aldol condensation. Additional reactions including a phosphate elimination reaction, isomerization, cyclization, and dehydration all operate in a single active site of MfnB [34]. This strategy is very rare in enzyme catalysis. Previous biochemical characterization of MfnB showed that two lysines and two aspartic acids around the active site are all essential for enzyme catalysis [34]. Of those, Lys27 forms a Schiff base during catalysis [34]. Combined with structural analysis, molecular docking, and biochemical characterization, two potential binding sites for GA-3P molecules in the active site were predicated and a catalytic mechanism was proposed (Figure 4). In the proposed mechanism, a phosphate elimination reaction and a isomeration reaction occurs at the GA-3P binding site I and II, respectively, prior to the aldol condensation between the enzyme-bound intermediates, after which the catalytic cycle is completed by a cyclization and two dehydration reactions assisted by several general acids/bases at the same active site [34]. However, this mechanism failed to assign the functions of each residue around the active site. Therefore, further uncovering the molecular basis of this catalytic mystery and understanding the structure-function relationship of MfnB will help in applying the knowledge to enzyme engineering in the future.

## 4. HMF toxicity

The carbohydrate that is released from hydrolysis of lignocellulose as a feedstock represents an alternative to produce biofuels and chemicals. However, the use of lignocellulose faces many challenges. Lignocellulosic hydrolysates produce many chemicals as byproducts produced from the hydrolysis process, such as aliphatic acids, furfural (furfural and 5-HMF), aromatic compounds, which can be toxic for the microbial fermentation at following steps. It has been reported that furfural and HMF containing aldehyde groups hinder growth and fermentation performance of *Escherichia coli* (*E. coli*) and *Saccharomyces cerevisiae* strains [46],[47]. Several possible toxic mechanisms have been identified and proposed: 5-HMF and furfural inhibit growth by damaging DNA, inhibiting glycolytic enzyme, chemically reacting with cellular components, decreasing the availability of NADPH [48]–[52]. However, the detailed toxic mechanism is still not completely understood.

*S. cerevisiae* and *E. coli* are capable of using multiple NAD(P)H-dependent oxidoreductases to convert furfural and HMF into less toxic alcohols [53]–[55], which are then excreted into culture medium and fermentation broth during fermentation. Without further degradation, however, the accumulation of furfuryl alcohol can eventually lead to growth inhibition. Some strains of *S. cerevisiae* and *E. coli* have been genetically engineered to better cope with elevated levels of HMF. A review paper has summarized the effort and progress to increase the *S. cerevisiae* and *E. coli* resistant to furfural and HMF [56]. Obviously, understanding the details of toxic mechanism will be beneficial to engineer *E. coli* and *S. cerevisiae* to be the excellent biocatalyst to mass-produce bulk chemicals and biofuels.

## 5. Microbial degradation of HMF and other furanic compounds

Few microorganisms show high concentration 5-HMF tolerance due to the presence of aerobic furfural and/or HMF degradation pathway. These furan-degrading microorganisms have been isolated and studied when growing them on HMF/furfural as sole carbon source medium, which have been summarized in a review paper [57]. Most furanic compound-degrading microorganisms belonging to a relatively small number of genera, aerobic gram-negative bacteria [57]. It cannot be excluded that furanic compounds can degrade via alternative pathways. For example, three fungal [58]–[60] and two anaerobic microorganisms [61],[62] have been reported to degrade furanic aldehyde as well. Thus, it is conceivable that characterization of furanic compounds in fungi or anaerobic bacteria will shine a light on the discovery of other furanic metabolism pathways.

**Figure 5.**
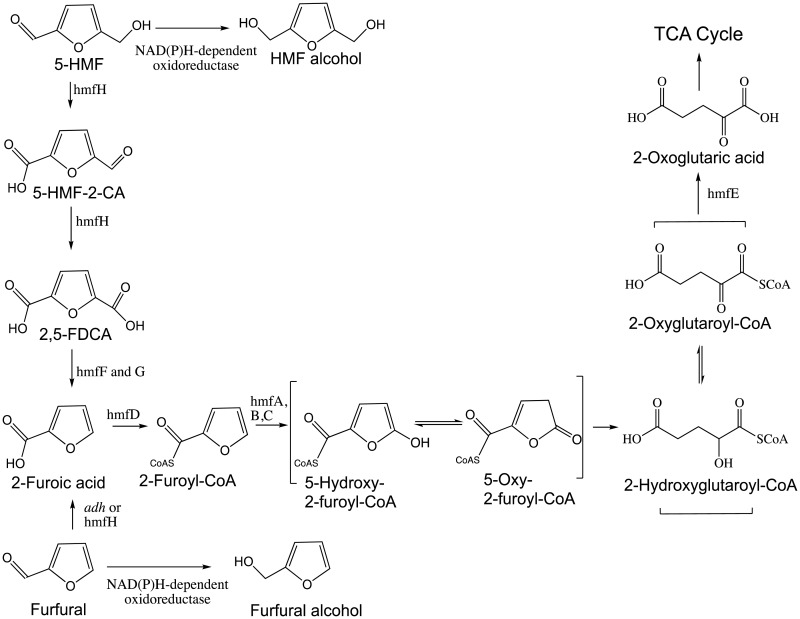
Degradative pathways of 5-HMF and furfual [57],[63].

The aerobic furfural degradation route present in *Pseudomonas putida* (*P. putida*) F2 was first proposed by P.W. Trudgill in 1969. The whole gene cluster responsible for degradation of furanic compounds was identified recently in Cupriavidus basilensis (*C. basilensis*) HMF14 that further confirms the “Trudgill pathway” (Figure 5) [63]. The “furfural cluster” contains the *hmfABCDE* genes, which are responsible for furfural degradation. In this pathway, furfural is first oxidized to 2-furoic acid by an aldehyde dehydrogenase (*adh*) or HMF oxidase (see below). 2-furoic acid is then subsequently ligated to coenzyme-A by a furoyl-CoA synthetase (hmfD). Then furoyl-CoA is hydroxylated at the C5 position by a furoyl-CoA dehydrogenase (hmfA, B and C). The resulting enol-CoA tautomerizes to its keto form, followed by the lactone ring opening through hydrolysis. After another keto-enol tautomerization, 2-oxoglutaroyl-CoA is formed. Through hydrolysis of the CoA thioester, 2-oxoglutarate is released (catalyzed by oxoglutaryl-CoA hydrolase, hmfE). 2-Oxoglutaric acid then can be metabolized through entering the tricarboxylic acid cycle [57],[63].

The *hmfFGH* gene cluster involved in the metabolism of HMF has also been elucidated [63]. 5-HMF is first oxidized to 2,5-FDCA by HMF oxidase (the gene product of *hmfH)* in two sequential steps, in which the alcohol group is first oxidized to the corresponding aldehyde, and then both aldehydes are further oxidized to carboxylic acids. FDCA is then decarboxylazed by furandicarboxylic acid decarboxylase (hmfF and hmfG) to 2-furoic acid, which it further processed through Trudgill pathway.

Genetic analysis indicated that the order of *hmfABCDE* gene is highly conserved; in contrast, the *hmfFGH* is much less conserved [57]. In addition to two gene clusters, additional genes might also be involved in degradation of HMF or furfural [57]. For example, *adh* gene encodes aldehyde dehydrogenase and is found in the vicinity of *hmf* gene cluster in several species. The nonspecific aldehyde dehydrogenase oxidizes furfural and 5-(hydroxymethyl)furan-2-carboxylic acid (5-HMF-2-CA) to 2-furoic acid and 2,5-FDCA, respectively, which helps cells against a variety of toxic aldehydes [57]. Furthermore, in *E. coli* and yeast, multiple NAD(P)H-dependent oxidoreductases convert furfural and HMF into less toxic alcohols during anaerobic fermentation [53]–[55].

Although the two gene clusters involved in furfural and HMF degradation were elucidated, further biochemical study of each enzyme in the pathway is necessary in the future for implementing the entire pathway into a microorganism or engineering an individual enzyme to apply in the improvement of the bioabatement lignocellulosic hydrolysates process. The most well-studied enzyme in the pathway is HMF oxidase (hmfH). However, the detailed enzymatic characterization of other enzymes in the gene cluster of *hmfABCDE* and *hmfFGH* has not been reported yet. The HMF oxidase was identified as an FAD-dependent oxidase, which belongs to the glucose-methanol-choline (GMC) oxidoreductase family [63]–[65]. The N-terminal GMC domain (Pfam 00732) is involved in binding of FAD, whereas the second conserved domain C-terminal GMC (Pfam 05199) contains a strictly conserved histidine (H467) [65],[66]. A *hmfH* gene cloned from *methylovorus* sp. MP688 has been successfully expressed and purified from *E. coli* [64],[65]. It showed a wide range of substrate spectrum. The most identified substrates are aromatic compounds including furanic, phenylic and cinnamylic primary alcohols, but strikingly promiscuous on the rest part of the molecule next to the alcohol group [65],[66]. The crystal structure of hmfH exhibited the similar structural architecture with two globular domains (a FAD-binding domain and a smaller cap domain), as other GMC members [66]. The active site is located at a deep and narrow cleft, which is formed at the interface of two domains [66]. The residues at the active site provide an essential H-bonding interaction that positions the substrate alpha carbon in direct contact with flavin and accommodates the aromatic moiety of the substrates through hydrophobic interactions with many hydrophobic residues [66]. Since hmfH catalyzes the oxidation of 5-HMF to 2,5-FDCA, which can be esterified to form polymers with a wide range of application [67]. Therefore, further understanding the structure-function relationships of hmfH will pave a way for future protein engineering aimed at special or improved biocatalytic properties.

Although the entire furfural degradation pathway has been discovered [63], it has not been introduced into common industrial hosts such as *E. coli* and *S. cerevisiae*. The obvious limitation for this pathway is its oxygen requirement. However, most industrial fermentation processes to produce bulk chemicals and biofuels are performed under anaerobic/micro-aerobic conditions. Therefore, it is expected to discover alternative metabolic pathways that can be applied in the industrial fermentation process.

## References

[1] Bozell JJ, Petersen GR (2010). Technology development for the production of biobased products from biorefinery carbohydrates—the US Department of Energy's “Top 10” revisited. Green Chem.

[2] Werpy T, Petersen G (2004). Top Value Added Chemicals from Biomass, National Renewable Energy Laboratory: Golden, CO.

[3] Zhang D, Dumont MJ (2017). Advances in polymer precursors and bio-based polymers synthesized from 5-hydroxymethylfurfural. J Polym Sci Pol Chem.

[4] Deng J, Pan T, Xu Q (2013). Linked strategy for the production of fuels via formose reaction. Sci Rep.

[5] Rosatella AA, Simeonov SP, Frade RFM (2011). 5-Hydroxymethylfurfural (HMF) as a building block platform: Biological properties, synthesis and synthetic applications. Green Chem.

[6] Cui MS, Deng J, Li XL (2016). Production of 4-Hydroxymethylfurfural from derivatives of biomass-derived glycerol for chemicals and polymers. ACS Sustain Chem Eng.

[7] van Putten RJ, van der Waal JC, de Jong ED (2013). Hydroxymethylfurfural, a versatile platform chemical made from renewable resources. Chem Rev.

[8] Yu IKM, Tsang DCW (2017). Conversion of biomass to hydroxymethylfurfural: A review of catalytic systems and underlying mechanisms. Bioresource Technol.

[9] Qin YZ, Zong MH, Lou WY (2016). Biocatalytic upgrading of 5-Hydroxymethylfurfural (HMF) with levulinic acid to HMF levulinate in biomass-derived solvents. ACS Sustain Chem Eng.

[10] Bohre A, Dutta S, Saha B (2015). Upgrading furfurals to drop-in biofuels: An overview. ACS Sustain Chem Eng.

[11] Caes BR, Teixeira RE, Knapp KG (2015). Biomass to furanics: Renewable routes to chemicals and fuels. ACS Sustain Chem Eng.

[12] Alexandrino K, Millera Á, Bilbao R (2014). Interaction between 2,5-dimethylfuran and nitric oxide: Experimental and modeling study. Energ Fuel.

[13] Zhong S, Daniel R, Xu H (2010). Combustion and emissions of 2,5-dimethylfuran in a direct-injection spark-ignition engine. Energ Fuel.

[14] Ray P, Smith C, Simon G (2017). Renewable green platform chemicals for polymers. Molecules.

[15] Burgess SK, Leisen JE, Kraftschik BE (2014). Chain mobility, thermal, and mechanical properties of poly(ethylene furanoate) compared to poly(ethylene terephthalate). Macromolecules.

[16] Papageorgiou GZ, Tsanaktsis V, Bikiaris DN (2014). Synthesis of poly(ethylene furandicarboxylate) polyester using monomers derived from renewable resources: thermal behavior comparison with PET and PEN. Phys Chem Chem Phys.

[17] Codou A, Moncel M, van Berkel JG (2016). Glass transition dynamics and cooperativity length of poly(ethylene 2,5-furandicarboxylate) compared to poly(ethylene terephthalate). Phys Chem Chem Phys.

[18] Dimitriadis T, Bikiaris DN, Papageorgiou GZ (2016). Molecular dynamics of poly(ethylene-2,5-furanoate) (PEF) as a function of the degree of crystallinity by dielectric spectroscopy and calorimetry. Macromol Chem Phys.

[19] Lomelí-Rodríguez M, Martín-Molina M, Jiménez-Pardo M (2016). Synthesis and kinetic modeling of biomass-derived renewable polyesters. J Polym Sci Pol Chem.

[20] Terzopoulou Z, Tsanaktsis V, Nerantzaki M (2016). Thermal degradation of biobased polyesters: Kinetics and decomposition mechanism of polyesters from 2,5-furandicarboxylic acid and long-chain aliphatic diols. J Anal Appl Pyrol.

[21] Baba Y, Hirukawa N, Tanohira N (2003). Structure-based design of a highly selective catalytic site-directed inhibitor of Ser/Thr protein phosphatase 2B (Calcineurin). J Am Chem Soc.

[22] Clark DE, Clark KL, Coleman RA (2005). Patent No. WO2004067524.

[23] Ermakov S, Beletskii A, Eismont O (2015). Brief review of liquid crystals. Liquid Crystals in Biotribology.

[24] Dewar MJS, Riddle RM (1975). Factors influencing the stabilities of nematic liquid crystals. J Am Chem Soc.

[25] Kowalski S, Lukasiewicz M, Duda-Chodak A (2013). 5-hydroxymethyl-2-furfural (HMF)—heat-induced formation, occurrence in food and biotransformation—a review. Pol J Food Nutr Sci.

[26] Murkovic M, Bornik MA (2007). Formation of 5-hydroxymethyl-2-furfural (HMF) and 5-hydroxymethyl-2-furoic acid during roasting of coffee. Mol Nutr Food Res.

[27] Murkovic M, Pichler N (2006). Analysis of 5-hydroxymethylfurfual in coffee, dried fruits and urine. Mol Nutr Food Res.

[28] Saha B, Abu-Omar MM (2014). Advances in 5-hydroxymethylfurfural production from biomass in biphasic solvents. Green Chem.

[29] Rout PK, Nannaware AD, Prakash O (2016). Synthesis of hydroxymethylfurfural from cellulose using green processes: A promising biochemical and biofuel feedstock. Chem Eng Sci.

[30] Mukherjee A, Dumont MJ, Raghavan V (2015). Review: Sustainable production of hydroxymethylfurfural and levulinic acid: Challenges and opportunities. Biomass Bioenerg.

[31] Thiyagarajan S, Pukin A, van Haveren J (2013). Concurrent formation of furan-2,5- and furan-2,4-dicarboxylic acid: unexpected aspects of the Henkel reaction. RSC Adv.

[32] Corre C, Song L, O'Rourke S (2008). 2-Alkyl-4-hydroxymethylfuran-3-carboxylic acids, antibiotic production inducers discovered by Streptomyces coelicolor genome mining. Proc Natl Acad Sci USA.

[33] Sidda JD, Corre C (2012). Gamma-butyrolactone and furan signaling systems in *Streptomyces*. Method Enzymol.

[34] Wang Y, Jones MK, Xu H (2015). Mechanism of the enzymatic synthesis of 4-(Hydroxymethyl)-2-furancarboxaldehyde-phosphate (4-HFC-P) from Glyceraldehyde-3-phosphate catalyzed by 4-HFC-P synthase. Biochemistry.

[35] Miller D, Wang Y, Xu H (2014). Biosynthesis of the 5-(Aminomethyl)-3-furanmethanol moiety of methanofuran. Biochemistry.

[36] Wang Y, Xu H, Jones MK (2015). Identification of the final two genes functioning in methanofuran biosynthesis in Methanocaldococcus jannaschii. J Bacteriol.

[37] Jia J, Schorken U, Lindqvist Y (1997). Crystal structure of the reduced Schiff-base intermediate complex of transaldolase B from *Escherichia coli*: mechanistic implications for class I aldolases. Protein Sci.

[38] Hester G, Brenner-Holzach O, Rossi FA (1991). The crystal structure of fructose-1,6-bisphosphate aldolase from *Drosophila melanogaster* at 2.5 A resolution. FEBS Lett.

[39] Sygusch J, Beaudry D, Allaire M (1987). Molecular architecture of rabbit skeletal muscle aldolase at 2.7-A resolution. Proc Natl Acad Sci USA.

[40] Blom N, Sygusch J (1997). Product binding and role of the C-terminal region in class I D-fructose 1,6-bisphosphate aldolase. Nat Struct Biol.

[41] Izard T, Lawrence MC, Malby RL (1994). The three-dimensional structure of N-acetylneuraminate lyase from *Escherichia coli*. Structure.

[42] Kim CG, Yu TW, Fryhle CB (1998). 3-Amino-5-hydroxybenzoic acid synthase, the terminal enzyme in the formation of the precursor of mC7N units in rifamycin and related antibiotics. J Biol Chem.

[43] Kim H, Certa U, Dobeli H (1998). Crystal structure of fructose-1,6-bisphosphate aldolase from the human malaria parasite *Plasmodium falciparum*. Biochemistry.

[44] Bobik TA, Morales EJ, Shin A (2014). Structure of the methanofuran/methanopterin-biosynthetic enzyme MJ1099 from *Methanocaldococcus jannaschii*. Acta Crystallogr F.

[45] Heine A, DeSantis G, Luz JG (2001). Observation of covalent intermediates in an enzyme mechanism at atomic resolution. Science.

[46] Almeida JRM, Röder A, Modig T (2008). NADH- vs NADPH-coupled reduction of 5-hydroxymethyl furfural (HMF) and its implications on product distribution in Saccharomyces cerevisiae. Appl Microbiol Biot.

[47] Palmqvist E, Hahn-Hägerdal B (2000). Fermentation of lignocellulosic hydrolysates. II: inhibitors and mechanisms of inhibition. Bioresource Technol.

[48] Modig T, Lidén G, Taherzadeh MJ (2002). Inhibition effects of furfural on alcohol dehydrogenase, aldehyde dehydrogenase and pyruvate dehydrogenase. Biochem J.

[49] Barciszewski J, Siboska GE, Pedersen BO (1997). A mechanism for the in vivo formation of N6-furfuryladenine, kinetin, as a secondary oxidative damage product of DNA. FEBS Lett.

[50] Horváth IS, Taherzadeh MJ, Niklasson C (2001). Effects of furfural on anaerobic continuous cultivation of Saccharomyces cerevisiae. Biotechnol Bioeng.

[51] Palmqvist E, Hahn-Hägerdal B (2000). Fermentation of lignocellulosic hydrolysates. I: inhibition and detoxification. Bioresource Technol.

[52] Nicolaou SA, Gaida SM, Papoutsakis ET (2010). A comparative view of metabolite and substrate stress and tolerance in microbial bioprocessing From biofuels and chemicals, to biocatalysis and bioremediation. Metab Eng.

[53] Wang X, Miller EN, Yomano LP (2011). Increased furfural tolerance due to overexpression of NADH-dependent oxidoreductase FucO in Escherichia coli strains engineered for the production of ethanol and lactate. Appl Environ Microb.

[54] Liu ZL, Blaschek HP (2010). Biomass conversion inhibitors andin situ detoxification. Biomass to Biofuels: Strategies for Global Industries.

[55] Liu ZL, Moon J, Andersh BJ (2008). Multiple gene-mediated NAD(P)H-dependent aldehyde reduction is a mechanism of in situ detoxification of furfural and 5-hydroxymethylfurfural by Saccharomyces cerevisiae. Appl Microbiol Biot.

[56] Nieves LM, Panyon LA, Wang X (2015). Engineering sugar utilization and microbial tolerance toward lignocellulose conversion. Front Bioeng Biotechnol.

[57] Wierckx N, Koopman F, Ruijssenaars HJ (2011). Microbial degradation of furanic compounds: biochemistry, genetics, and impact. Appl Microbiol Biot.

[58] Zhang J, Zhu Z, Wang X (2010). Biodetoxification of toxins generated from lignocellulose pretreatment using a newly isolated fungus, Amorphotheca resinae ZN1, and the consequent ethanol fermentation. Biotechnol Biofuels.

[59] Trifonova R, Postma J, Ketelaars JJMH (2008). Thermally treated grass fibers as colonizable substrate for beneficial bacterial inoculum. Microbial Ecol.

[60] López MJ, Nichols NN, Dien BS (2004). Isolation of microorganisms for biological detoxification of lignocellulosic hydrolysates. Appl Microbiol Biot.

[61] Boopathy R, Daniels L (1991). Isolation and characterization of a furfural degrading sulfate-reducing bacterium from an anaerobic digester. Curr Microbiol.

[62] Brune G, Schoberth SM, Sahm H (1983). Growth of a strictly anaerobic bacterium on furfural (2-furaldehyde). Appl Environ Microb.

[63] Koopman F, Wierckx N, de Winde JH (2010). Identification and characterization of the furfural and 5-(hydroxymethyl)furfural degradation pathways of *Cupriavidus basilensis* HMF14. Proc Natl Acad Sci USA.

[64] Dijkman WP, Groothuis DE, Fraaije MW (2014). Enzyme-catalyzed oxidation of 5-hydroxymethylfurfural to furan-2,5-dicarboxylic acid. Angew Chem Int Edit.

[65] Dijkman WP, Fraaije MW (2014). Discovery and characterization of a 5-Hydroxymethylfurfural oxidase from *Methylovorus* sp. strain MP688. Appl Environ Microb.

[66] Dijkman WP, Binda C, Fraaije MW (2015). Structure-based enzyme tailoring of 5-hydroxymethylfurfural oxidase. ACS Catal.

[67] de Jong E, Dam MA, Sipos L (2012). Furandicarboxylic acid (fdca), a versatile building block for a very interesting class of polyesters. Biobased Monomers, Polymers, and Materials.

